# Clinical outcomes of Botox injection according to subgroups of esophagogastric junction outflow obstruction: A single institutional retrospective analysis

**DOI:** 10.1097/MD.0000000000043005

**Published:** 2025-06-20

**Authors:** Geon Woo Lee, Su Jin Kim, Cheol Woong Choi, Su Bum Park, Dae Gon Ryu, Woo Jin Kim

**Affiliations:** a Department of Internal Medicine, Pusan National University School of Medicine and Research Institute for Convergence of Biomedical Science and Technology, Pusan National University Yangsan Hospital, Yangsan, South Korea.

**Keywords:** botulinum toxins, esophagogastric junction, manometry

## Abstract

The Chicago Classification version 4.0 categorizes esophagogastric junction outflow obstruction (EGJOO) into 4 subgroups based on peristalsis patterns. Recent proposals introduce new terminology, grouping EGJOO with distal esophageal spasm and a hypercontractile esophagus as major mixed motility disorders (MMMDs), while classifying ineffective esophageal motility and normal peristalsis as isolated or ineffective esophagogastric junction outflow obstruction (IEGJOO). Botulinum toxin (Botox) injection is considered a cost-effective, minimally invasive treatment option for EGJOO. This study aimed to investigate clinical outcomes of Botox injection based on these subgroups. We included all patients over 18 years old who underwent high-resolution manometry at our institution between May 2019 and December 2023. Patients diagnosed with EGJOO and treated with Botox injections were categorized into subgroups. Clinical outcomes were assessed using Eckardt scores (ESs) at diagnosis and 2 months posttreatment. Among 180 patients, 31 met the Chicago Classification 4.0 criteria for EGJOO, and 22 of these received Botox injections. Six of these patients had MMMD, and 16 had ineffective esophagogastric junction outflow obstruction. MMMD showed a higher distal contractile integral, but no other significant differences in high-resolution manometry were observed. Patients with MMMD had higher posttreatment ESs (5.50 [2.75–6.25] vs 2.00 [1.00–2.75]; *P* = .009) and lower changes in ESs (1.00 [0.75–2.50] vs 4.00 [2.25–4.75]; *P* =.010) when compared to those with IEGJOO. This study suggests that Botox injection is less effective in treating MMMD compared to IEGJOO, which may impact treatment strategies for different EGJOO subgroups.

## 1. Introduction

**Figure 1.**
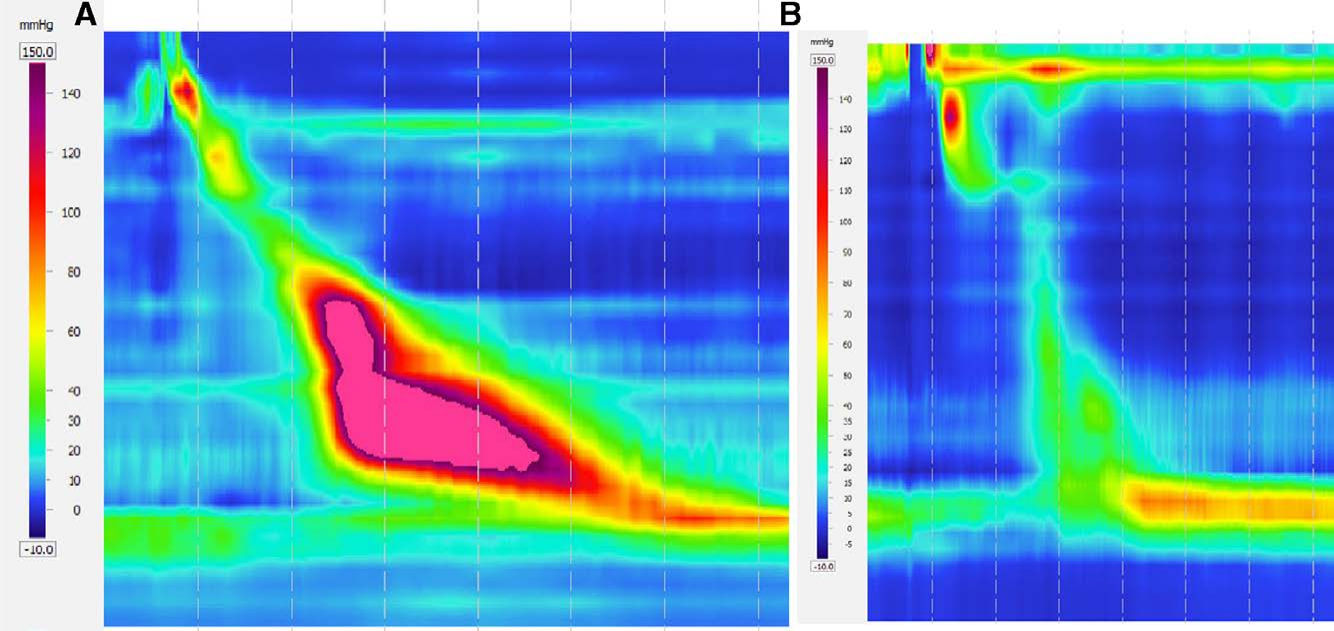
HRM examples of EGJOO subgroups (A) EGJOO with hypercontractile features. (B) EGJOO with ineffective motility. EGJOO = esophagogastric junction outflow obstruction, HRM = high-resolution manometry,.

Esophagogastric junction outflow obstruction (EGJOO) is a motility disorder characterized by impaired relaxation of the esophagogastric junction, quantified by an elevation of integrated relaxation pressure (IRP), with preserved esophageal peristalsis.^[1,2]^ To reduce the number of clinically irrelevant diagnoses and prevent unnecessary treatment, the Chicago Classification version 4.0 (CCv4) introduced a rigorous diagnostic criteria for EGJOO. Additionally, the CCv4 defines 4 subgroups of EGJOO based on peristalsis patterns: EGJOO with spastic features, EGJOO with hypercontractile features, EGJOO with ineffective motility, and EGJOO with no evidence of disordered peristalsis^[2]^ (Fig. 1). Recently, a new terminology has been proposed, grouping EGJOO with distal esophageal spasm and hypercontractile esophagus (HE) as major mixed motility disorder (MMMD) and those with minor disorders of peristalsis such as ineffective esophageal motility and normal peristalsis as isolated or ineffective esophagogastric junction outflow obstruction (IEGJOO). This classification categorizes EGJOO into 2 subgroups based on characteristic patterns of peristalsis.^[3]^

Treatment options for EGJOO include medications such as smooth muscle relaxants, calcium channel blockers, nitrates, and tricyclic antidepressants and procedures such as botulinum toxin (Botox) injection, pneumatic dilation, and myotomy (peroral endoscopic myotomy [POEM], Heller myotomy).^[4–8]^ Medications are generally not considered first-line therapy owing to varying response rates (0–75%).^[7,9–11]^ Endoscopic procedures aim to reduce the pressure of the lower esophageal sphincter (LES). According to a systematic review, Botox injection at the LES has shown a favorable response rate of 58%, comparable to that seen in achalasia.^[6,9,12–17]^ However, this response rate is lower than the 93% clinical success that was achieved with POEM at 6 months post-procedure.^[18]^ Despite its relatively lower efficacy, Botox injection remains a preferred option at our institution because it is an easy, cost-effective, safe, and minimally invasive intervention.^[19,20]^

In this study, we examined previously diagnosed cases of EGJOO based on the CCv4. We compared symptoms, high-resolution manometry (HRM) findings, and responses to Botox injection between patients categorized into MMMD and IEGJOO subgroups. We aimed to investigate the differences in clinical outcomes following Botox injection among EGJOO subgroups.

## 2. Materials and methods

### 2.1. Patients and data selection

The study protocol was approved by the Institutional Review Board of our hospital, confirming that the study was conducted in accordance with the ethical guidelines (Institutional Review Board No. 55-2024-087). Informed consent was waived by the ethics committee because the patients’ medical records were anonymized prior to analysis. This study was carried out according to the ethical guidelines outlined in the Declaration of Helsinki. As a retrospective study, patients older than 18 years who underwent HRM at our hospital between May 2019 and December 2023 were identified from a maintained clinical database. According to the CCv4 criteria, the diagnosis of EGJOO is established when there is an elevated IRP in both supine (>15 mm Hg) and upright (>12 mm Hg) positions, accompanied by obstructive symptoms such as dysphagia or noncardiac chest pain. Additionally, adjunctive tests such as timed barium esophagogram (TBE) or an endoscopic functional lumen imaging probe should demonstrate findings suggestive of EGJOO.^[1,2]^ At our institution, we utilized TBE, where the criteria for diagnosing EGJOO is defined as >4 cm of retained liquid barium in the esophagus at 1 minute.^[3]^ Exclusion criteria encompassed secondary EGJOO, patients not meeting CCv4 criteria, or those who received treatments other than Botox injection (e.g., medications, pneumatic dilation) or no treatment.

Patients finally diagnosed with EGJOO who underwent Botox injection were classified into subgroups based on esophageal peristalsis patterns as follows: EGJOO with spastic features if ≥20% swallows with premature/spastic contraction were observed, EGJOO with hypercontractile features if ≥20% hypercontractile swallows were observed, EGJOO with ineffective motility if >70% ineffective swallows or ≥50% failed peristalsis was observed, and EGJOO with normal peristalsis if no evidence of disordered peristalsis was found.^[2]^ The subgroups demonstrating hypercontractile or spastic features were collectively termed MMMD, while the remaining 2 groups were classified as IEGJOO.^[3]^ To increase accuracy of classification, 2 gastroenterologists skilled in HRM interpretation worked together.

Clinical outcomes were assessed using the Eckardt score (ES) for dysphagia, chest pain, regurgitation, and weight loss both at diagnosis and 2 months posttreatment. Symptoms were evaluated using a scale from 0 to 3: 0 for no symptoms, 1 for occasional symptoms, 2 for daily symptoms, and 3 for symptoms occurring with each meal. Weight loss was categorized as follows: 0 for no weight loss, 1 for <5 kg lost, 2 for 5 to 10 kg lost, and 3 for more than 10 kg lost. Clinical improvement was defined as a posttreatment ES of <3 or improvement in ES by ≥1 when baseline ES was <3.

### 2.2. HRM protocol

All HRM procedures were performed using computerized HRM analysis software (Manoview, Medtronic) following the standardized protocol of our institution. The procedure begins with the patient in the supine position. After catheter placement, a 60-second rest is allowed for adaptation, followed by confirmation of the catheter position using at least 3 deep breaths. A 30-second baseline period is then recorded to identify anatomical landmarks such as the upper esophageal sphincter, LES, respiratory inversion point, and basal EGJ pressure. Following this, ten 5-mLwet swallows of room temperature saline are performed, ensuring 30-second intervals between swallows to avoid deglutitive inhibition. The patient is then repositioned to an upright position. After another 60-second adaptation period and catheter confirmation via deep breaths, a second 30-second baseline period is recorded to reidentify landmarks. At least five 5 mL wet swallows are performed within the same 30-second interval. If the results remain inconclusive or if there is suspicion of EGJ outflow obstruction not meeting achalasia criteria, a TBE is conducted to further assess for obstruction.^[2]^

### 2.3. Endoscopic injection technique of Botox

A total of 100 units of Botox A were administered endoscopically within 1 cm proximal to the squamocolumnar junction, targeting areas with strong contractions. The injection was divided into 4 aliquots, each containing 25 units of Botox, delivered to individual quadrants while maintaining a perpendicular orientation to the esophageal wall.^[19–21]^ When performing the procedure at an oblique angle rather than vertically, there is a risk of submucosal injection, potentially resulting in a focal submucosal bleb. Additionally, if the needle is inserted too deeply, the injection may extend beyond the esophageal wall. These factors should be carefully considered during endoscopic procedures for successful Botox injection.

### 2.4. Statistical analysis

Categorical variables are presented as frequencies and percentages, while numerical variables are expressed as median or mean ± standard deviation. Statistical differences between groups were assessed using the Kruskal–Wallis, Mann–Whitney *U*, and chi-square tests, with a 95% confidence interval calculated. A 2-tailed probability value (*P*-value) < .05 was considered statistically significant. All statistical analyses were performed using SPSS (Statistical Package for the Social Science; SPSS, Inc., Chicago) version 15 for Microsoft Windows.

## 3. Results

**Figure 2.**
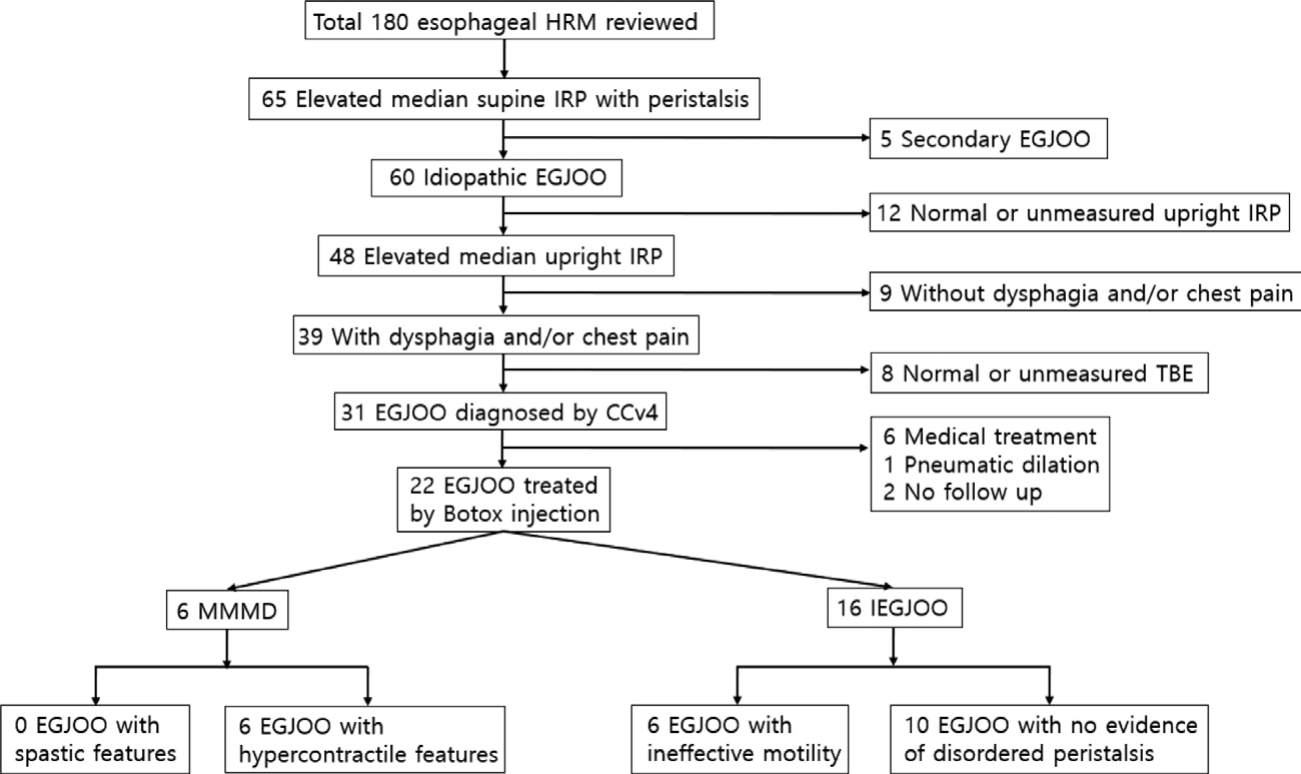
Distribution of patients with EGJOO treated with Botox injection into subgroups. Botox = botulinum toxin, CCv4 = Chicago Classification version 4.0, EGJOO = esophagogastric junction outflow obstruction, HRM = high-resolution manometry, IEGJOO = ineffective esophagogastric junction outflow obstruction, IRP = integrated relaxation pressure, TBE = timed barium esophagogram.

Among 180 patients who underwent HRM at our institution, five were excluded due to secondary causes of EGJOO, such as bariatric surgery, esophageal cancer, benign esophageal stricture, extraluminal compression from an epiphrenic diverticulum, or an aortic aneurysm. No patient used opioids. Additionally, 12 patients were excluded because of normalization of IRP in the upright position or incomplete IRP measurement. Nine patients did not present with typical symptoms like dysphagia or chest pain, and 8 patients had normal adjunctive TBE results or were not tested. Of all patients, 31 (17.2%) met the CCv4 criteria for EGJOO; among those with elevated supine IRP, 47.7% exhibited this characteristic. As primary treatment, 22 patients received Botox injections, 6 received medications, 1 underwent pneumatic dilation, and 2 did not follow up after diagnosis. Among the 22 patients who received Botox injections, 13 experienced clinical improvement, while 9 did not. Of those 9, 2 underwent pneumatic dilation as a follow-up treatment, and 7 chose to continue with medication therapy, declining further procedures. Additionally, within the Botox injection group, 6 patients had MMMD, and 16 had IEGJOO. None of the MMMD patients exhibited spastic features, while all 6 showed hypercontractile features. Within the IEGJOO group, 6 exhibited ineffective motility, and 10 showed no peristalsis disorder (Fig. 2**).**

Comparing patient characteristics between the MMMD and IEGJOO groups, there were no significant differences observed in age (66.67 ± 3.22 vs 65.19 ±3.95 years; *P* = 1.000), sex (33.3% female vs 56.3% female; *P* = .635), height (164.67 ± 3.15 vs 159.88 ± 2.25 cm; *P* = .184), weight (64.00 ± 4.52 vs 58.69 ± 2.59 kg; *P* = .172), and body mass index (23.62 ± 1.51 vs 22.91 ± 0.82 kg/m^2^; *P* = .338). In the HRM data, excluding distal contractile integral (10,523.93 ± 3032.68 vs 1488.32 ± 310.64 mm Hg s cm; *P <* .001), there were no significant differences in median supine IRP (32.65 ± 5.12 vs 30.25 ± 2.74 mm Hg; *P* = .685), median upright IRP (33.62 ± 7.49 vs 27.71 ± 2.62 mm Hg; *P* = .580), mean intrabolus pressure (38.88 ± 2.86 vs 34.55 ± 2.48 mm Hg; *P* = .294), and distal latency (7.35 ± 0.41 vs 7.97 ± 0.63 seconds; *P* = .768) between MMMD and IEGJOO (Table 1**).**

**Table 1:** Comparison of patient profile and high-resolution manometry data between major mixed motility disorder and ineffective esophagogastric junction outflow obstruction.

	MMMD	IEGJOO	*P*-value
Patient profile			
N	6	16	
Age (yr)	66.67 ± 3.22	65.19 ± 3.95	1.000
Sex (% female)	33.3%	56.3%	.635
Height (cm)	164.67 ± 3.15	159.88 ± 2.25	.184
Weight (kg)	64.00 ± 4.52	58.69 ± 2.59	.172
BMI (kg/m^2^)	23.62 ± 1.51	22.91 ± 0.82	.338
HRM data			
IRP supine (mm Hg)	32.65 ± 5.12	30.25 ± 2.74	.685
IRP upright (mm Hg)	33.62 ± 7.49	27.71 ± 2.62	.580
IBP (mm Hg)	38.88 ± 2.86	34.55 ± 2.48	.294
DCI (mm Hg·cm s)	10,523.93 ± 3032.68	1488.32 ± 310.64	<.001
DL (s)	7.35 ± 0.41	7.97 ± 0.63	.768

BMI = body mass index, DCI = distal contractile integral, DL = distal latency, HRM = high-resolution manometry, IBP = intrabolus pressure, IEGJOO = ineffective esophagogastric junction outflow obstruction, IRP = integrated relaxation pressure, MMMD = major mixed motility disorder.

When comparing clinical outcomes before and after Botox treatment, the results showed better clinical improvement in patients with IEGJOO than in those with MMMD (16.7% vs 75.0%; *P* = .015). There were no significant differences in median pretreatment ES (6.00 [5.50–7.50] vs 6.00 [5.00–6.75]; *P* = .398). Two months after Botox injection, the MMMD group showed a higher posttreatment ES compared to the IEGJOO group (5.50 [2.75–6.25] vs 2.00 [1.00–2.75]; *P* = .009), and the change in ES was greater in the IEGJOO group (1.00 [0.75–2.50] vs 4.00 [2.25–4.75]; *P* = .010; Table 2).

**Table 2:** Clinical outcomes of Botox injection in major mixed motility disorder and ineffective esophagogastric junction outflow obstruction.

	MMMD	IEGJOO	*P*-value
% of clinical improvement after Botox injection	16.7%	75.0%	.015
Pretreatment ES	6.00 [5.50–7.50]	6. [5.00–6.75]	39,800
Posttreatment ES	5.50 [2.75–6.25]	2.00 [1.00–2.75]	.009
Changes in ES	1.00 [0.75–2.50]	4.00 [2.25–4.75]	.010

Botox = botulinum toxin, ES = Eckardt score, IEGJOO = ineffective esophagogastric junction outflow obstruction, MMMD = major mixed motility disorder.

Additionally, in our study, the most reported primary symptom before the procedure was dysphagia (86.4%), followed by chest pain (13.6%). However, there were no significant differences in treatment effectiveness between the 2 groups (*P* = .527).

## 4. Discussion

Unlike achalasia, a disease with well-established pathophysiology, diagnosis, classification, and treatment, EGJOO is a motility disorder that is relatively less understood and under investigation through various research efforts. Some studies have shown that many patients with EGJOO exhibit abnormal esophageal body motility.^[22,23]^ Subsequently, in CCv4, EGJOO was categorized into 4 subgroups based on peristalsis patterns.^[2]^ Additionally, Leopold et al^[3]^ grouped similar subgroups and proposed 2 distinct subsets of EGJOO: MMMD and IEGJOO.

The pathophysiology of EGJOO remains incompletely understood, but it is hypothesized to involve the loss of inhibitory nerve fibers akin to achalasia.^[7,24]^ While EGJOO is often considered a variant or an early-stage form of achalasia, most cases do not progress to achalasia.^[9,15,25]^ Moreover, distinct differences in neuroimmunological profiles suggest that EGJOO may represent a unique clinical entity separate from achalasia.^[26]^ Unlike IEGJOO, MMMD is characterized by a hypercontractile or spastic esophageal body, attributed to an excess of cholinergic drive and temporal asynchrony of circular and longitudinal muscle contractions.^[11,27–29]^

The natural course of EGJOO is unpredictable and can vary widely, with reports of spontaneous resolution.^[9,11,17]^ Therefore, particularly for patients with mild symptoms, it may be wise to first consider conservative approaches like close observation or Botox injection to the LES before pursuing definitive treatments that permanently alter LES tone.^[5–8]^

Botox, derived from the anaerobic fermentation of *Clostridium botulinum*, is classified as type A among various forms of botulinum toxins. As a potent inhibitor of acetylcholine release at the neuromuscular junction, Botox can prevent LES contraction, thereby reducing LES pressure and facilitating improved esophageal flow dynamics.^[19,20]^ However, the benefits of Botox injection were not sustained over the long term. This is due to the development of neuronal sprouts from motor nerve terminals, which reestablish synaptic activity after Botox inhibits synaptic vesicle fusion.^[20,30]^ In our study, after Botox injection in patients with EGJOO, symptomatic improvement was observed in 59.1% of them at 2 months, which is similar to the findings in previous literature.^[6,9,12–17]^ However, this rate declined to 40.9% at 3 months and to 31.8% at 4 months. Therefore, we recommend evaluating the treatment response at least monthly for 3 months after Botox injection. If there is insufficient improvement, it may be worth considering retreatment with Botox or exploring other procedures such as pneumatic dilation or POEM.

Our study investigated the short-term treatment effects of Botox injection in EGJOO subgroups, specifically MMMD and IEGJOO. Our findings suggest that patients with MMMD have a lower response to Botox therapy compared to those with IEGJOO when measured by ES. Despite reducing IRP by decreasing LES pressure through Botox treatment, the residual peristaltic dysfunction in the esophageal body contributes to less pronounced symptom relief after treatment. Schupack et al conducted a long-term follow-up study of patients with EGJOO and HE. They found that higher maximal distal contractile integral and IRP at diagnosis were associated with prolonged symptoms such as dysphagia and chest pain (*P* < .05). This supports the notion that symptoms in EGJOO and HE arise from dysfunctional esophageal peristalsis and increased bolus transit resistance through the gastroesophageal junction.^[11]^

The current study is constrained by its small sample size and retrospective design. Additionally, repeat HRM studies and timed barium swallows were not conducted to assess improvements in EGJOO, and the short-term follow-up duration limits conclusions about intervention durability and long-term outcomes. Therefore, the data should be interpreted cautiously, and further research with larger sample sizes, prospective designs, and extended follow-up is needed to validate and expand upon our findings.

In summary, when EGJOO is diagnosed according to the stringent criteria of the CCv4, selecting the appropriate treatment is crucial. Botox injection is considered a first-line treatment due to its minimally invasive, safe, and cost-effective nature compared to pneumatic dilation or POEM. Our research indicated that the effectiveness of Botox treatment varies depending on the subgroup of EGJOO, with MMMD showing less favorable outcomes than IEGJOO. Despite decreased LES pressure after Botox treatment in MMMD, the persistence of hypercontractile or spastic features in esophageal peristalsis could contribute to ongoing symptoms. EGJOO involves not only elevated LES pressure but also consideration of the peristalsis pattern in the esophageal body. Therefore, it is essential to approach treatment differently based on EGJOO subgroups.

## Author contributions

**Conceptualization:** Geon Woo Lee, Su Jin Kim.

**Data curation:** Geon Woo Lee, Su Jin Kim.

**Methodology:** Cheol Woong Choi, Su Bum Park, Dae Gon Ryu, Woo Jin Kim.

**Supervision:** Cheol Woong Choi, Su Bum Park, Dae Gon Ryu.

**Writing – original draft:** Geon Woo Lee.

**Writing – review & editing:** Geon Woo Lee, Woo Jin Kim.
